# Complete mitochondrial genome of freshwater fish, *Microphysogobio jeoni* (Actinopterygii, Cypriniformes, Cyprinidae), from South Korea

**DOI:** 10.1080/23802359.2020.1715276

**Published:** 2020-01-27

**Authors:** Philjae Kim, Jeong-Ho Han, Seung Lak An

**Affiliations:** a Research Division, National Science Museum, Daejeon, Korea;; b Water Environmental Management Department, Korea water Resources Corporation, Daejeon, Korea

**Keywords:** Freshwater fish, complete mitochondrial genome, *Microphysogobio jeoni*, phylogeny, Gobioninae

## Abstract

In this study, we determined the complete mitochondrial genome sequences of cyprinid freshwater fish, *Microphysogobio jeoni*, belonging to the subfamily Gobioninae in the order Cypriniformes. The complete mitogenome of *M. jeoni* was 16,602 bp in length and consisted of 13 protein-coding genes (PCGs), 22 tRNAs, and two rRNAs. The gene order was identical with other *Microphysogobio* species. The overall nucleotide composition of *M. jeoni* was A + T: 56.1% and G + C: 43.9%, with slightly AT bias. In the phylogenetic tree, *M*. *jeoni* and other congeneric species clearly formed a monophyletic clade, and each species distinguished against each other well.

Genus *Microphysogobio* Mori, 1934 belonging to Cyprinidae inhabits in Korea peninsula, China, North Vietnam, Laos, Japan, and Mongolia (Huang et al. 2017). A total of 28 species of the genus *Microphysogobio* have been known worldwide, and five species reported as Korean endemic species. These five species have presently sequenced entire mitochondrial genomes, except *Microphysogobio jeoni* and endangered species *M. rapidus* (Tang et al. 2011; Hwang et al. 2014; Park et al. 2016).

In this study, the complete mitochondrial DNA sequence (MN581867) of *M*. *jeoni* was first examined using NGS analysis. *M*. *jeoni* was collected in Baengma-River, Buyeo-gun, South Korea (36°17′33.04″N, 126°54′32.07″E) on 21 June 2019 and identified by morphology (Son and Song 2006). The specimen was deposited in the Natural History Laboratory of National Science Museum (Daejeon, Korea). The mitochondrial DNA (mt-DNA) was isolated from caudal fin using the Qproteome^®^ Mitochondria Isolation Kit (QIAGEN, Hilden, Germany) and DNeasy Blood & Tissue DNA isolation kit (QIAGEN) (stored in −20 °C until use). The mt-DNA was amplified using REPLI-g Mitochondrial DNA kit (QIAGEN). A sequencing library was constructed from the mitochondrial DNA using a QIAseq FX single cell DNA library kit (QIAGEN) with paired end reading, followed by sequencing on the Illumina Hi-Seq 2500 platform (San Diego, CA, USA).

The complete mitogenome sequence of *M*. *jeoni* (MN581867) was 16,602 bp in length and contained 13 PCGs, 22 tRNA genes, and two rRNA genes. The gene order was exactly same as other *Microphysogobio* species. ND6 and eight tRNA genes were encoded on light strand, and the others on heavy strand. The overall nucleotide compositions were 30.0% A, 26.1% T, 26.8% C, and 17.1% G. All PCGs have initiation codons, ‘ATG’, except for COX1 started with ‘GTG’. The terminal codon of five PCGs (ND2, COX1, ATP8, ATP6, and ND4L) is ‘TAA’ and three PCGs (ND1, ND5, and ND6) have ‘TAG’. The three PCGs (COX2, COX3, and CytB) and the rest of PCGs (ND3 and ND4) have incomplete stop codon ‘T––’ and ‘TA–’, respectively.

To examine the phylogenetic relationship between *Microphysogobio* and other Cypriniformes taxa, mitogenome of *M*. *jeoni* was analyzed with 15 of Cypriniformes. Two Siluriformes species, *Silurus asotus* (JX256247) and *Liobagrus mediadiposalis* (KR075136) were used as outgroups. The 13 PCGs and two rRNAs were included in dataset. The best-fit substitution model was estimated using jModelTest 2.1.1 (Guindon and Gascuel 2003). For ML analysis, PhyML 3.1 with GTR + I + G model was used and performed using rapid option with 1000 iterations bootstrap resampling (Guindon et al. 2010).

In the phylogenetic analysis result, *M*. *jeoni* was grouped distinctly from the congeneric species. These species were grouped with other taxonomical species of Cyprinidae. Also, this clade was clearly divided with Acheilognathidae and formed larger clade, Cypriniformes (Figure 1). As a result, the complete mitogenome obtained in this study will be a genomic resource for evolution studies of genus *Microphysogobio*.

**Figure 1.**
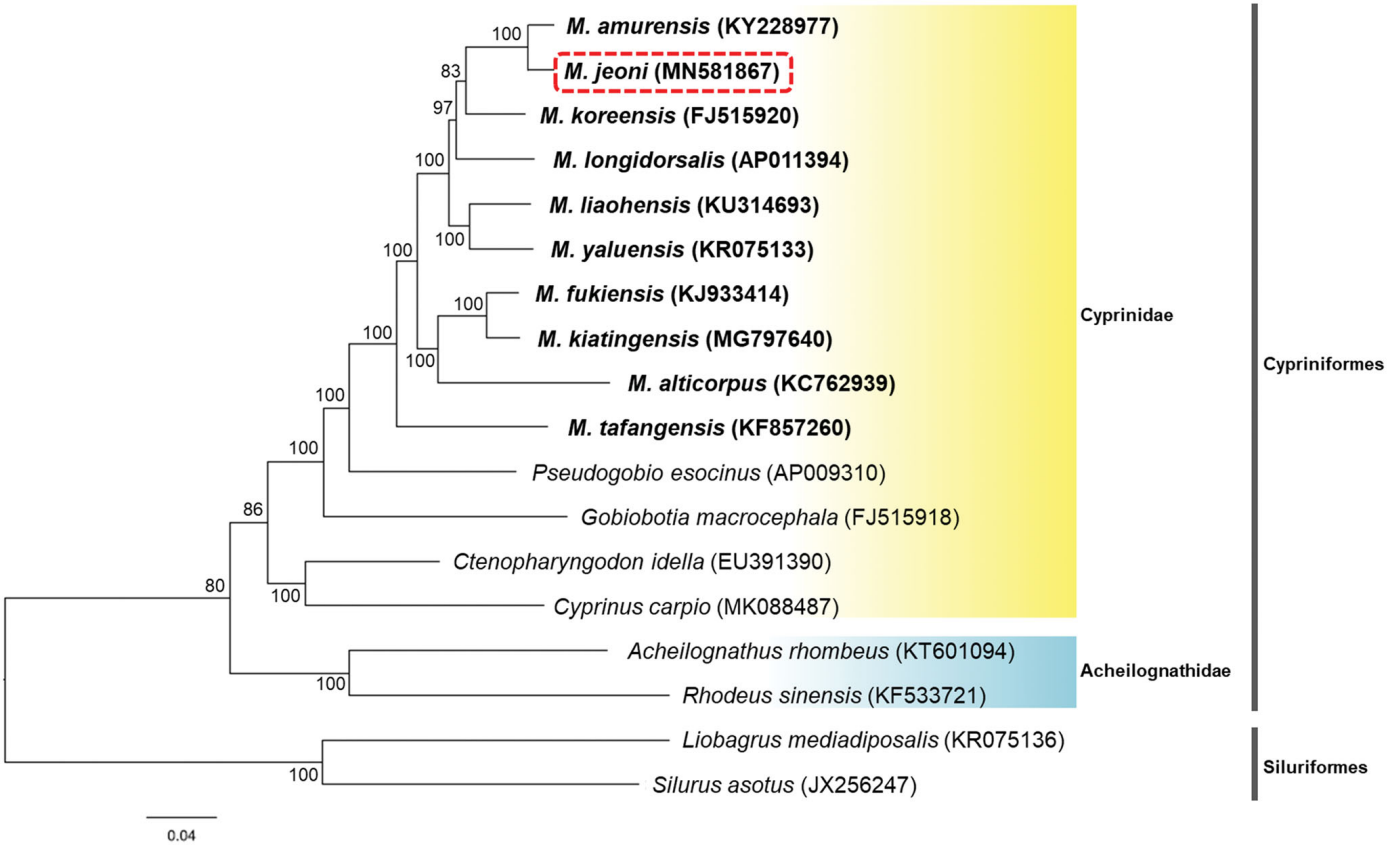
Phylogenetic tree of maximum-likelihood (ML) method based on the nucleotide sequences of 13 PCGs and two rRNAs of 15 cyprinids, included *M*. *jeoni* (MN581867), and two catfishes. Bootstrap support values are indicated on each node as >70.
